# The Impact of COronaVIrus Disease of 2019 Pandemic on Home Health Nursing Availability in Pediatric Patients After Tracheostomy Placement

**DOI:** 10.1002/ppul.71273

**Published:** 2025-09-05

**Authors:** Ferdinand Cacho, Katherine A. Carr, Mariana Bedoya, Jacob A. Kaslow

**Affiliations:** ^1^ Department of Pediatrics, Division of Pediatric Allergy, Immunology and Pulmonary Medicine Vanderbilt University Medical Center Nashville Tennessee USA; ^2^ Department of Pediatrics Washington University School of Medicine, Division of Allergy and Pulmonary Medicine St. Louis Missouri USA

**Keywords:** covid‐19, home health, nursing, tracheostomy

## Abstract

**Background:**

Children with tracheostomies require skilled medical care performed by trained caregivers or home health nursing (HHN). HHN services are often limited, resulting in increased caregiver responsibilities. We aim to evaluate HHN availability, healthcare utilization, and mortality in tracheostomy dependent children, pre and post‐COVID‐19 pandemic.

**Methods:**

Retrospective chart review of pediatric patients who underwent tracheostomy between November 2017 and September 2023. The COVID‐19 pandemic time point was defined as March 15, 2020. Demographic, clinical and outcome parameters were collected from the electronic medical record. The pre‐ and postpandemic trend of HHN hours were evaluated using correlation statistics.

**Results:**

Both groups had similar surrogate markers of medical complexity. There was no difference in the mean number of approved HHN hours, but both the mean number of HHN hours/week staffed (90 h vs. 50, *p* = 0.001) and mean percentage of approved HHN hours staffed (63% vs. 41%, *p* = 0.011) were significantly lower in the post‐COVID‐19 cohort. The trend in the pre‐COVID‐19 group was not statistically significant (*ρ* = 0.087, *p* = 0.639); the trend in the post‐COVID‐19 group was negative (*ρ* = −0.380, *p* = 0.001).

**Conclusions:**

Since the COVID‐19 pandemic, there has been a decrease in both the number and percentage of HHN hours staffed with no change in the number of approved HHN hours. Additionally, there was a decrease in hospital encounters in the post‐COVID‐19 group compared to pre‐COVID‐19. By assessing workforce shifts, medical providers, caregivers, and policymakers can appropriately anticipate the impact on this vulnerable population.

## 1 Introduction

As the survivability of complex cardiopulmonary and neurologic conditions in neonates and children has improved, the number of tracheostomy dependent children has increased [1]. Tracheostomy is estimated to be performed in 0.2% of pediatric inpatient stays [2] and in over 4800 children under 2 years of age each year [3]. Children with tracheostomies require skilled medical care in the home setting including tracheostomy tube replacement and cleaning, stoma care, frequent suctioning, delivery of inhaled medications and airway clearance techniques, management of humidification, often times mechanical ventilation, and emergency tracheostomy care. Given the complex medical care required for children with tracheostomies in the home setting and high potential for life‐threatening complications, extensive caregiver training including both decision making and technical skills is required. Despite skilled training and educational programs for caregivers, tracheostomy‐related complications are common with a high incidence of emergency department (ED) visits, subsequent hospitalization within 90 days postdischarge, mean healthcare costs and mortality compared to the general population [4, 5].

A 2016 official statement released by the American Thoracic Society about the outpatient care of a child with a tracheostomy, recommends an awake and attentive trained caregiver be in the home of a child requiring chronic invasive ventilation at all times [6]. However, these guidelines highlight that caring for these children in the home environment “can rarely be accomplished without the use of professional caregivers.” Additionally, many children with tracheostomies are discharged without home health nursing [7] (HHN) despite the nearly universal sentiment amongst healthcare providers that patients receive far fewer nursing hours than they should receive [8]. This is due to a myriad of factors including ubiquitous home care nursing shortages, which have been shown to correlate with prolonged hospital length of stay (LOS) and readmission for children with medical complexity [9, 10, 11, 12]. Since the COronaVIrus Disease of 2019 (COVID‐19) pandemic, home healthcare nursing shortages have worsened and both new patients and existing patients have experienced lapses in home nursing support [13, 14]. This decrease is augmented by numerous challenges unique to home pediatric care such as nursing comfort with medical complexity, variable training practices [15], and inconsistent hiring/staffing [16]. With families reporting a variety of issues such as financial hardship [17], difficulty completing daily household tasks [18], disruptions to mental well‐being [19, 20], and significant time burden [21], there is increased importance of resources to alleviate caregiver stressors.

In this study, we aim to evaluate the effect of COVID‐19 on HHN availability, emergency department and hospital readmission rates and mortality in tracheostomy dependent children at our pediatric medical center.

## 2 Methods

We performed a retrospective review from November 2017 to September 2023 at our tertiary referral pediatric hospital located in the Southeast United States with a catchment area across a 200 mile radius, spanning seven states. Our patient population includes urban, suburban and rural families across a wide spectrum of socioeconomic statuses. The inclusion criteria were all pediatric patients who underwent tracheostomy placement during the time frame. The exclusion criteria included death during the admission in which tracheostomy was placed, and if the patient had not yet been discharged from tracheostomy placement admission at the time of analysis. Demographic parameters included age of tracheostomy, sex, race, and ethnicity. Clinical data included respiratory severity score (RSS = mean airway pressure × fraction of inspired oxygen) at the time of discharge after tracheostomy placement (as a marker of severity of respiratory illness [22]), degree of respiratory support at the time of discharge after tracheostomy placement, complex care provider involvement and LOS after tracheostomy placement were abstracted from the electronic medical record (EMR) by chart review. The COVID‐19 pandemic time point was defined as March 15, 2020 and this date was used to classify participants into two groups: pre‐COVID‐19 and post‐COVID‐19. Outcome variables collected from the EMR included approved HHN hours, HHN hours staffed, and respiratory related hospital encounters (defined as emergency department visits or hospital readmission) within 30 days, 3 months and 6 months after discharge. Hospital encounters were determined to be respiratory related based on chief complaint and encounter diagnosis by chart review. The percentage of approved HHN staffed hours was calculated by dividing staffed hours by approved hours.

Chi‐square and two‐sample *t*‐tests or Mann–Whitney *U*‐tests were performed where appropriate to assess differences in outcome and clinical variables between the pre‐COVID‐19 and post‐COVID‐19 groups. To explore the trend of HHN hours over time, the proportion of approved HHN staffed hours were plotted as a time series. Spearman's rank correlation was calculated for each group's trend line.

The study was approved by the Vanderbilt University Medical Center Institutional Review Board and informed consent was not required for this retrospective study conducted via analysis of existing previously collected data. This manuscript was written with the guidance of guidelines for reporting observational studies (The Strengthening the Reporting of Observational Studies in Epidemiology [STROBE]) [23].

## 3 Results

A total of 143 children underwent tracheostomy placement during the study period. One hundred and twenty‐six subjects met inclusion criteria. Baseline demographic characteristics from the study population are listed in Table 1. Thirty‐five participants (28%) were discharged before the COVID‐19 pandemic and categorized into the pre‐COVID‐19 group and 91 participants (72%) were discharged after the start of the COVID‐19 pandemic and were placed in the post‐COVID‐19 group. Sex, race, and age at tracheostomy were similar between the two groups (Table 1).

**Table 1:** Demographic characteristics of the study cohort.

	Study population (*n* = 126)	Pre‐COVID‐19 (*n* = 35)	Post‐COVID‐19 (*n* = 91)	*p* value
Male sex	82 (65%)	22 (63%)	60 (66%)	0.746
Race				0.242
White	78 (62%)	18 (50%)	60 (68%)	
Black	30 (24%)	11 (31%)	19 (22%)	
Asian	5 (4%)	3 (8%)	2 (2%)	
Hispanic/Latino	9 (7%)	3 (8%)	6 (7%)	
Other	2 (2%)	0 (0%)	2 (2%)	
Age at tracheostomy in days				0.717
Mean (SD)	1387 (2166)	1273 (2068)	1430 (2212)	
Median (IQR)	256 (132, 1480)	193 (80, 1964)	294 (145, 1426)	

*Note:* For categorical variables, data are presented as *n* (%). For continuous variables, data are presented as mean (SD) and median (IQR).

There were no statistically significant clinical differences in LOS after tracheostomy placement (*p *= 0.271), RSS (*p *= 0.667), degree of respiratory support (*p *= 0.488), and complex care provider involvement (*p *= 0.888) at the time of hospital discharge after tracheostomy placement between the pre‐COVID‐19 and post‐COVID‐19 cohort (Table 2).

**Table 2:** Clinical characteristics of the pre‐COVID‐19 and post‐COVID‐19 groups at the time of discharge after tracheostomy placement.

	Pre‐COVID‐19 (*n* = 35)	Post‐COVID‐19 (*n* = 91)	*p* value
Hospital length of stay after tracheostomy in days			0.271
Mean (SD)	65 (43)	87 (113)	
Median (IQR)	62 (28, 94)	62 (28, 97)	
Respiratory severity score	3.7 (2.5, 4.7)	3.2 (2.3, 4.9)	0.667
Respiratory support			0.488
Full ventilator > 20 h	18 (55%)	44 (52%)	
Full ventilator during sleep	1 (3%)	8 (10%)	
CPAP/BiPAP > 20 h	1 (3%)	1 (1%)	
CPAP/BiPAP during sleep	2 (6%)	4 (5%)	
Oxygen only	1 (3%)	0 (0%)	
Room air only	10 (30%)	27 (32%)	
Followed by complex care	23 (66%)	61 (67%)	0.888

*Note:* For categorical variables, data are presented as *n* (%). For continuous variables, data are presented as mean (SD) and median (IQR).

**Figure 1:**
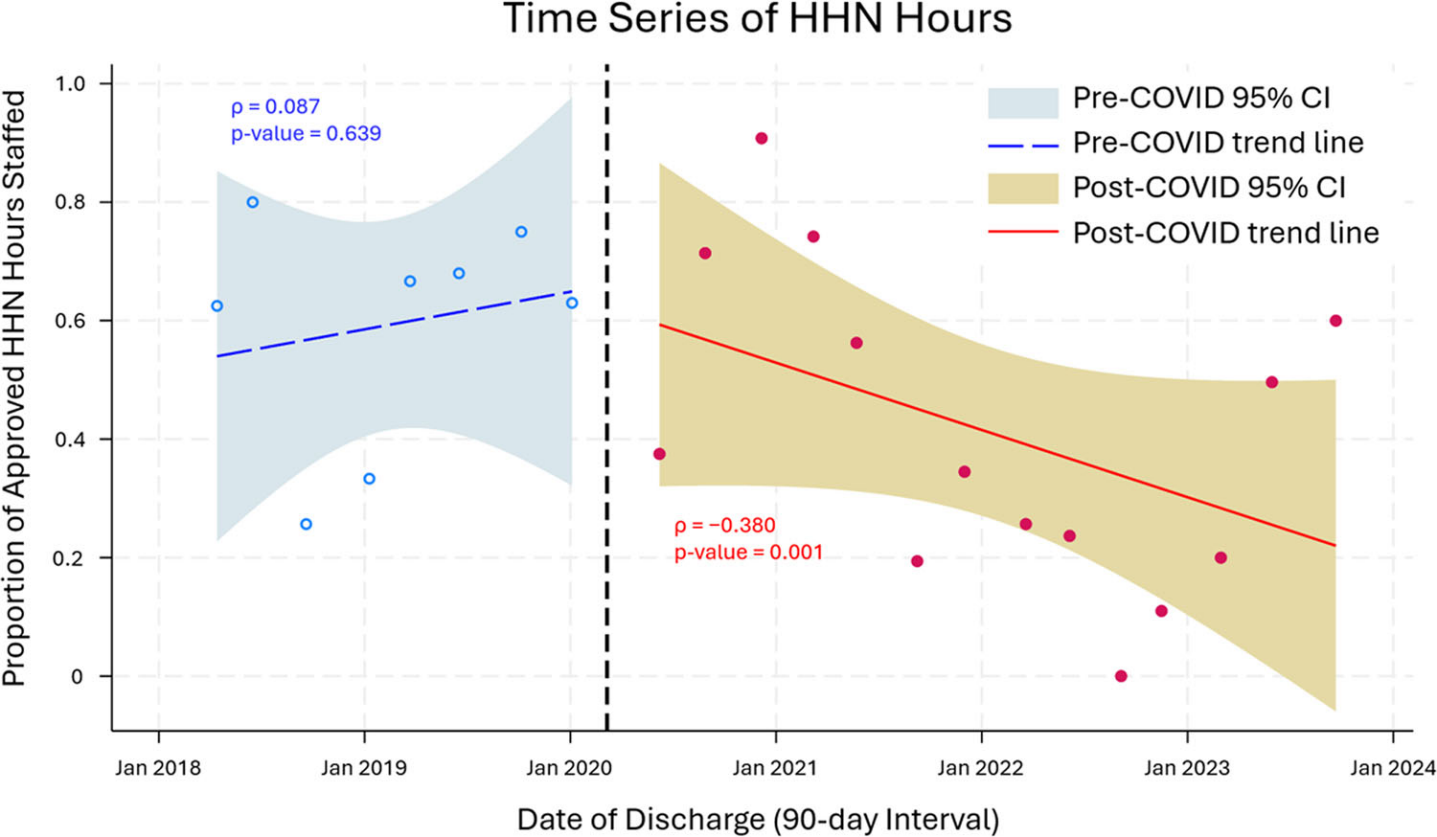
Time series of home health nursing (HHN) hours. The proportion of approved HHN staffed hours are plotted by time of discharge in 90‐day average intervals. Vertical reference line corresponds to March 15, 2020, the start of the COVID‐19 pandemic. Spearman's rank correlation in the pre‐COVID‐19 group was not statistically significant (*ρ* = 0.087, *p* = 0.639); the correlation in the post‐COVID‐19 group was negative (*ρ* = −0.380, *p* = 0.001). [Color figure can be viewed at wileyonlinelibrary.com]

In the pre‐COVID‐19 group, at time of discharge, the mean number of insurance‐approved HHN hours per week was 142, the mean number of HHN hours staffed per week was 90 with an average percentage of approved hours staffed of 63% (Table 3). In the post‐COVID‐19 group, the mean number of insurance approved HHN hours per week was 120, the mean number of HHN hours per week staffed was 50 with an average percentage of approved hours staffed of 41%. When comparing these two groups, there was no statistically significant difference in the number of weekly HHN hours approved (*p *= 0.068), but both the number of staffed HHN hours and percentage of approved HHN staffed hours were significantly lower in the post‐COVID‐19 cohort (*p *= 0.001 and *p* = 0.011, respectively). The proportion of approved HHN staffed hours were represented as a time series, plotted by time of discharge in 90‐day average intervals (Figure 1). The trend in the pre‐COVID‐19 group was not statistically significant (*ρ* = 0.087, *p* = 0.639) while the post‐COVID‐19 group trend was negative (*ρ* = −0.380, *p* = 0.001).

**Table 3:** Home health nursing (HHN) per week and clinical outcomes pre and post‐COVID‐19 pandemic.

	Pre‐COVID‐19 (*n* = 35)	Post‐COVID‐19 (*n* = 91)	*p* value
HHN hours approved per week			0.068
Mean (SD)	142 (46)	120 (59)	
Median (IQR)	168 (126, 168)	168 (84, 168)	
HHN hours staffed per week			0.001
Mean (SD)	90 (68)	50 (54)	
Median (IQR)	84 (36, 168)	44 (0, 84)	
Proportion of approved HHN hours staffed			0.011
Mean (SD)	0.63 (0.38)	0.41 (0.37)	
Median (IQR)	0.60 (0.36, 1.0)	0.36 (0, 0.75)	
Readmission/ED within 30 days of discharge	13 (41%)	22 (26%)	0.121
Readmission/ED within 3 months of discharge	21 (66%)	38 (45%)	0.044
Readmission/ED within 6 months of discharge	24 (75%)	45 (53%)	0.031
Death	5 (16%)	8 (9%)	0.329

*Note:* For categorical variables, data are presented as *n* (%). For continuous variables, data are presented as mean (SD) and median (IQR).

Of the pre‐COVID‐19 group, 13 (41%) children had hospital encounters within 30 days of discharge, 21 (66%) had hospital encounters within 3 months, and 24 (75%) had hospital encounters within 6 months of discharge after tracheostomy placement. The post‐COVID‐19 group had 22 (26%) hospital encounters within 30 days, 38 (45%) hospital encounters within 3 months, 45 (53%) hospital encounters within 6 months of discharge after tracheostomy placement. When comparing outcomes between these two groups, the pre‐COVID‐19 group had higher overall rates of postdischarge hospital encounters (*p* = 0.031). Within the study period, there were 5 (16%) deaths in the pre‐COVID‐19 cohort compared to 8 (9%) deaths in the post‐COVID‐19 cohort (*p* = 0.37) postdischarge.

## 4 Discussion

This retrospective analysis of discharges from a single institution suggests that since the COVID‐19 pandemic, there has been a significant decrease in both the number and percentage of staffed HHN hours. Despite this acute decrease in HHN availability, we did not identify an increase in hospital readmission rates, ED visits or mortality.

Our observation of decreased staffed HHN hours supports previously published data surrounding a national nursing shortage exacerbated by the COVID‐19 pandemic with one analysis showing the total supply of registered nurses decreased by more than 100,000 in the year 2021 alone, demonstrating the greatest decrease over the past four decades [24]. Other pandemic‐driven disruptions such as medication supply and family caregiving interruptions have been reported by caregivers of pediatric patients with medical complexity [14, 25, 26, 27, 28]. However, this study focuses on the vulnerable sub‐population of tracheostomy‐dependent children and to our knowledge, HHN availability since the COVID‐19 pandemic has not been reported in this population.

While our study population did not show an increase in healthcare utilization or mortality in the post‐COVID‐19 pandemic group compared to the pre‐COVID‐19 pandemic group, this secondary outcome was a numerical count of healthcare utilization encounters and did not assess the severity of the reason of admission. The pre‐ and post‐COVID‐19 cohorts did not have significant differences in terms of surrogate measures of medically complexity with no statistically significant differences in RSS at discharge, the degree of required respiratory support at discharge, LOS after tracheostomy placement and the number of patients followed by the complex care team. This suggests that healthcare utilization and mortality were not necessarily influenced by medical complexity as assessed by surrogate markers. However, we did find a noticeable difference between these groups, with the post‐COVID‐19 cohort receiving significantly decreased HHN support.

While the overwhelming majority of providers within the United States report an inadequate supply of home nurses [29], the nursing shortage in the United States is expected to worsen over the next several years with a projected a total national deficit of 918,232 registered nurse jobs by the year 2030 [30]. This shortage will remain a factor in clinical decision making for these patients with the presence of HHN often dictating discharge planning [31] with recent publications reporting that lack of home care nursing is the most common cause of discharge delay [32], accounting for an average increase in LOS of 53.9 days [10]. Our findings may be limited by the small sample size which may not have been powered to detect a significant difference between clinical outcomes, including LOS. Studies have shown an increased LOS during the pandemic with a decrease in availability of a trained caregiver [33]. Decreasing LOS may result in significant cost savings for both the patient and hospital system.

It is important to note that there are several other factors other than medical safety and cost that may influence a provider's comfort level with proceeding with discharge in the absence of HHN. Our study did not address important nonclinical outcomes that may be affected by home nursing availability, such as caregiver quality of life and caregiver missed workdays. Without HHN, parents and family members are forced to assume the primary medical care role although the presence of HHN does provide a different set of challenges for caregivers [19]. Qualitative studies investigating the burden associated with families performing complex medical care at home cite several adverse effects including lack of sleep, depression, low self‐esteem, financial stress related to healthcare expenditure, extensive time spent coordinating care and navigating the complex healthcare system, practical and psychological constraints in spending time outside the home and negative impacts on siblings [19, 21, 34, 35, 36]. Additional factors that may play a role in candidacy for discharge without HHN in the tracheostomy population are socioeconomic status, proximity to a healthcare center, housing accommodations, additional children in the home and number of caregivers. Even though our data did not find an association between lack of HHN and healthcare utilization or mortality, caregiver and social factors that may be affected by lack of HHN must also be heavily considered when discussing discharge candidacy after tracheostomy placement.

There are several limitations to our study. This represents a retrospective, single center design with a relatively small sample size. In more generalized studies with longer time periods and larger number of patients, the presence of HHN has been shown to be associated with reduced readmission rates, fewer number of days in the hospital and overall lower hospital costs [11]. Our study may have been underpowered to detect significant differences. Further time series or trend analytics were not performed due to the small sample size. Additionally, there are many factors associated with the COVID‐19 pandemic itself that we did not control for in this study that could have influenced healthcare utilization and outcomes after discharge. One major possible factor is decreased viral exposure associated with staying home, patients or siblings not attending school in person, wearing masks in public, telemedicine visits, more outpatient antibiotic prescriptions for illness in attempts to avoid ED visits, etc [14]. With these modifications, it is theorized that children in the post‐COVID‐19 pandemic group developed fewer acute illnesses, contributing to decreased healthcare utilization. Another limitation is the heterogeneity of the pediatric population in regard to tracheostomy indication, with some patients having severe lung disease requiring continuous mechanical ventilation and others requiring tracheostomy for upper airway obstruction. Despite this heterogeneity, both groups had similar surrogate measures of medical complexity, so we do not suspect a significant effect on our outcome measures. Our study may have limited generalizability based on the single center design and the demographics of the study population.

Additional studies are needed to both qualify and quantify the impact of the COVID‐19 pandemic and HHN on both clinical and nonclinical outcomes. Ideally, future research will include input from multiple centers across a variety of regions with variable access to home health services to better characterize the significance of the decrease in HHN availability.

## 5 Conclusion

Since the COVID‐19 pandemic, there has been a significant decrease in both the number and percentage of staffed HHN hours in the pediatric patients who required placement of tracheostomy. Despite this decrease in HHN availability, our study did not find an increase in ED presentation rates, hospital readmission rate or mortality, consistent with decrease in hospital utilization among patients with a broad range of medical complexities who had a tracheostomy placed. The effect of decreased HHN availability on important nonclinical outcomes should be the focus of additional studies in the context of the current national home nursing shortage. By assessing workforce shifts, medical providers, caregivers, and policymakers can appropriately anticipate the impact on this vulnerable population.

## Author Contributions

**Ferdinand Cacho:** methodology, writing – review and editing, formal analysis, software. **Katherine A. Carr:** conceptualization, investigation, writing – original draft, writing – review and editing. **Mariana Bedoya:** writing – review and editing, investigation. **Jacob A. Kaslow:** conceptualization, investigation, writing – review and editing, methodology, data curation, supervision.

## Conflicts of Interest

The authors declare no conflicts of interest.

## Supporting information

supmat.

## Data Availability

The data that support the findings of this study are available from the corresponding author upon reasonable request.
